# Efficacy and tolerability of a novel herbal formulation for weight management in obese subjects: a randomized double blind placebo controlled clinical study

**DOI:** 10.1186/1476-511X-11-122

**Published:** 2012-09-20

**Authors:** Krishanu Sengupta, Atmatrana T Mishra, Manikeswar K Rao, Kadainti VS Sarma, Alluri V Krishnaraju, Golakoti Trimurtulu

**Affiliations:** 1 Laila Impex R&D Center, Unit-I, Phase-III, J. Autonagar, Vijayawada, India; 2 Department of Internal Medicine, ASR Academy of Medical Sciences, Eluru, India; 3 Suraksha Health Village, Gurunanak Nagar, Vijayawada, India; 4 Department of Statistics, SV University, Tirupati, India

**Keywords:** Adiponectin, Adipromin, Clinical trial, LI85008F, Obesity, Preadipocyte factor 1

## Abstract

**Background:**

The effect of an herbal formulation LI85008F on weight loss in obese human subjects was evaluated in an 8-weeks randomized, double-blind, placebo-controlled study (Clinical Trial Registration no. ISRCTN37381706). Fifty obese subjects (Body mass index 30 to 40 kg/m², 29.3% male; 70.7% female; ages 27–50 years) were randomized into two groups; placebo (n = 25) and LI85008F formulation (n = 25). The participants received either 900 mg/day of LI85008F formulation in three divided doses or three identical placebo capsules and all of them remained on a calorie-controlled diet (2000 cal/day) and 30 min walking for 5 days a week during the entire duration of the study.

**Results and discussion:**

At the end of the trial period, LI85008F supplemented group showed significant net reductions in body weight and Body Mass Index (BMI). The participants who received the herbal formulation, showed reduced fasting blood glucose, LDL, LDL/HDL ratio, and triglycerides. At the end of the study, LI85008F supplementation also provided 21.26% (p = 0.012) increase in serum adiponectin level, compared with the placebo group. No major adverse events were reported by the participants in the study duration. In addition, Adipokine profiling study in 3T3-L1 adipocytes demonstrates that LI85008F modulates key regulatory factors of adipogenic differentiation and insulin sensitivity, such as Adiponectin, Pref-1, and resistin.

**Conclusion:**

The herbal formulation LI85008F (Adipromin) is prepared from commonly used medicinal plants extracts, which provides useful and safe application for weight loss in obese humans. It also demonstrates potential promise in controlling healthy blood glucose level in obesity linked type 2 diabetes.

## Introduction

Obesity is a prevalent health hazard in developed and developing countries and is closely associated with various pathological disorders, including diabetes, hypertension, and cardiovascular diseases. Obesity arises from increased size of individual adipose cells due to lipid accumulation and from increased number of adipocytes arising from differentiation of adipose precursor cells to mature adipocytes under the appropriate nutritional and hormonal influences [1-3]. In a recent analysis jointly conducted by the International Association for the Study of Obesity (IASO), and the International Obesity Task Force (IOTF) in 2010, estimates that approximately 1.0 billion adults are currently overweight (BMI 25–29.9 Kg/m²), and a further 475 million are obese. Globally, IASO/IOTF also estimate that up to 200 million school aged children are either overweight or obese, and of those 40–50 million are classified as obese. In the European Union 27 member states, approximately 60% of adults and over 20% of school-age children are overweight or obese. This equates to around 260 million adults & over 12 million children being either overweight or obese [4].

Treatment of obesity includes lifestyle-based intervention (diet, exercise, and behavior therapy) and medical or surgical intervention (pharmacotherapy or bariatric surgery) [5]. There are several approaches through which pharmaco-therapies are directed to treat obesity. These include, limiting the absorption of food, suppressing appetite and reducing food intake, and altering metabolism or increasing energy expenditure [6]. Unfortunately, the use of pharmacological therapy remains controversial. These weight-loss medications are known to have significant adverse effects [7,8]. Therefore, the combination of safety concerns and high costs associated with weight-loss drugs is motivating majority of populations to continue to rely on traditional healing methods using the indigenous pharmacopoeia, such as plant based therapy.

Recently, we have shown that an herbal formulation, LI85008F also known as Adipromin has potent anti-adipogenic activity in mouse adipocytes *in vitro*[9]. LI85008F is comprised of the extracts of three medicinal plants, *Moringa olifera, Murraya koeingii and Curcuma longa*. Our observations suggested that LI85008F inhibited lipogenesis in adipocytes and concurrently antagonized PPARγ and other lipogenic factors. In addition, LI85008F enhanced triglyceride mobilization from the fat cells or promoted lipolysis [9]. Further, we also have demonstrated broad-spectrum safety of LI85008F in various animal models [10].

These interesting observations prompted us to evaluate the weight-loss promoting efficacy of LI85008F as primary outcome variable in obese humans in a placebo-controlled double blind clinical study. In this study, we also evaluated the short term safety of LI85008F and the secondary outcome variables such as, metabolic and cardiovascular parameters.

## Materials and methods

### Study material

LI85008F or Adipromin is an herbal formulation comprised of ethanol extract of *Moringa olefera* leaves, aqueous alcohol extract of *Murrya koenigii* leaves and ethanol extract of *Curcuma longa* rhizomes standardized to 95% total curcuminoids, mixed at a ratio of 6:3:1, respectively [9].

### Recruitment of subjects

**Figure 1:**
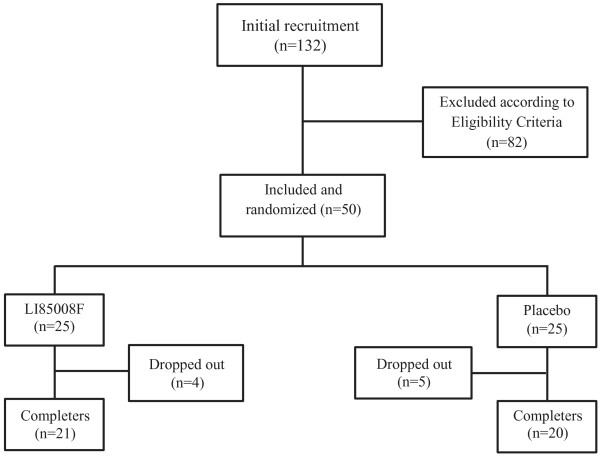
**Flow chart of the study design.** Evaluations of vital signs, body weight, BMI, serum biochemistry, hematology and urinalysis were done at baseline (day 0) and on 2, 4 and 8 weeks during follow up. Assessment of serum adiponectin level was done only on 8^th^ week.

This trial was performed at Alluri Sitarama Raju Academy of Medical Sciences (ASRAM), Eluru, Andhra Pradesh, India (clinical trial registration number: ISRCTN37381706). The study protocol was evaluated and approved by the ASRAM Institutional Review Board (IRB). An overview of the clinical study is provided in Figure 1. Briefly, in the preliminary phase screening, 132 subjects out of 285 attending the Internal Medicine Outpatients’ Department of the ASRAM Hospital were selected by a questionnaire based screening procedure. A total of 50 obese subjects (BMI 30 to 40 kg/m²) were included in the study through inclusion/exclusion criteria (Table 1), and each of them voluntarily signed the IRB approved informed consent. After recruitment, the subjects were randomly distributed into placebo and treatment groups. The demographic data and baseline characteristics are summarized in Table 2.

**Table 1:** Inclusion and Exclusion criteria

**Criteria**	**Details**
Inclusion	Subjects must understand risks and benefits of the protocol.
	Adults ages 21–50 years.
	BMI ≥30 kg/m².
	Willingness to participate in the exercise-walking program, Supervised by a trained exercise specialist.
	Willingness to consume the prescribed study diet of approximately 2,000 K Cal per day as outlined in the protocol (meals will be provided at free of coast by the study sponsor).
	Written informed consent to participate in the trail.
	Willingness to complete standard health history questionnaire before recruitment into the study.
	Willingness to participate in 5 clinical visits (Screening, baseline, 2, 4 & 8 weeks).
	If female and:Should be negative in pregnancy test.Of child bearing potential, should agree to follow an acceptable method of birth control for the duration of the study, such as condoms, foams, jellies, diaphragm, intrauterine device (IUD), etc.
Exclusion	History of thyroid disease or cardiovascular disease or diabetes.
	Any other clinically significant disorder.
	History of allergy to spices and herbal products.
	Non-obese (BMI < 30) & morbidly obese (>40).
	Presently using other weight loss medications, as well as stimulants, laxatives or diuretics taken solely for the purpose of weight loss.
	Pregnant or nursing females.
	Recent, unexplained weight loss or gain.
	Positive HIV test.
	History of hepatitis, pancreatitis, lactic acidosis or hepatomegaly with steatosis.
	History of motor weakness or peripheral sensory neuropathy.
	Any evidence of organ dysfunction or any clinically significant deviation from the normal, in physical or clinical determinations.

**Table 2:** Demographic data and baseline characteristics of the study subjects

**Characteristics**	**Placebo (n =20)**	**LI85008F (n = 21)**
Gender		
Men	5	7
Women	15	14
Age (Yrs, Mean ± SE))	37.2 ± 1.52	41.6 ± 1.37
Weight (Kg, Mean ± SE))	84.97 ± 2.80	86.16 ± 2.75
BMI (Kg/m², Mean ± SE))	33.0 ± 0.73	34.41 ± 0.74

### Study design

Based on the observations obtained from *in vitro* experiments in adipocytes [9]**,** we hypothesized that LI85008F might be useful for weight management in obese subjects. The primary study outcome was reduction in body weight and BMI. Sample size calculations were done using power analysis based on previous obesity study report [11]. More than 90% power at the two-tailed α level of 0.05 would be provided to test the significance of weight reduction over placebo with a minimum of 25 subjects per group.

After recruitment, the subjects were block randomized and included in active or placebo group (n = 25). All the study investigators were blinded; the clinical trial pharmacist and statistician ensured that the treatment codes remained confidential. Active or placebo capsules, compliance card and list of instructions including moderate exercise and 2000 cal standard diet and dates of follow up evaluations were provided to all the subjects at the baseline evaluation. Each active capsule contains 300 mg of LI85008F and 200 mg excipients. Each placebo capsule containing 500 mg of excipient is identical in appearance, size, weight and color. The subjects were advised to take 3 capsules a day; 30 min before breakfast, lunch and dinner.

All subjects filled a questionnaire, providing details regarding demographics, medical history and nutritional status at the baseline evaluation and during follow up evaluations at 14, 28 and 56 days. At the baseline evaluation and at each follow up visit during the 56 days period, all subjects were assessed for several anthropomorphic parameters such as body weight, height, waist circumference, hip circumference; vital signs and various parameters of serum biochemistry, hematology and complete urine examination. Blood and urine samples were collected at all evaluation days to measure various parameters including hematology, differential white blood cell count, biochemical markers and complete urinalysis.

### Hematological and biochemical evaluations

For an assessment of safety of LI85008F, several parameters were evaluated in serum, urine and whole blood of all subjects at each visit of the study duration. Serum biochemical parameters and hematological parameters were measured using the automated analyzer HumaStar 300 (Human, Wiesbaden, Germany) and the hematological counter Humacount (Human) respectively. The urine analysis was carried out using UroColor™10 Dip Sticks (Standard Diagnostics, Kyonggi-do, Korea) and by microscopy of sediment. Fasting blood glucose level was measured by enzymatic colorimetric method using a commercial kit (GLUCOSE liquicolor, Human, Weisbaden, Germany) following the protocol provided by the vendor.

### Serum adiponectin levels

Serum adiponectin levels were determined by specific EIA method by using human adiponectin ELISA kit (LINCO Research, St. Charles, MO).

### Adipokine array

Modulations of adipogenesis markers *in vitro* in LI85008F treated 3T3-L1 mouse adipocytes was evaluated by using a proteome profiler (R&D Systems, Minneapoplis, MN). Cell culture and treatments were performed following the method described earlier [9]. Briefly, 3T3-L1 mouse embryo fibroblasts were obtained from American Type Culture Collection (Manassas, VA) and cultivated in maintenance medium comprised of DMEM supplemented with 10% fetal bovine serum (FBS) 100 U/ml penicillin, 100 μg/ml streptomycin, 1 mM sodium pyruvate and 4.5 g/L D-glucose. Equal number of 3T3-L1 cells (60,000 cells per well) was seeded in each well of 24-well tissue culture plates and grown to confluence. Cells were allowed to differentiate for five days in presence or absence of 50 μg/ml LI85008F as described earlier [9]. The control cultures received only 0.1% (v/v) DMSO as the vehicle. Following treatment, the cells were washed and cell lysates was prepared in cell lysis buffer. The protein concentrations were estimated by Bradford reagent.

The Adipokine expression profiling was performed by using Adipokine proteome profiler (Mouse Adipokine Array kit, Catalog# ARY013, R&D Systems, Minneapoplis, MN), following the instructions provided by the vendor. Briefly, the analyte pre-coated membranes were blocked in blocking buffer for one hour at room temperature. Three hundred microgram protein samples and detection antibody mixture was further incubated with the pre-blocked membranes overnight at 4°C. Thereafter, the washed membranes were incubated with Streptavidin-HRP for 30 min at room temperature. Finally, the antigen-antibody reaction was detected by chemiluminescent detection substrate (Catalog No #34080 Thermo scientific, Rockford, IL) and the image was captured by a Molecular imager (ChemiDoc XRS+, BioRad, Hercules, CA). Densitometric analysis of immunoreactions on captured image was performed by Image Lab software version 2.0.1 (BioRad, Hercules, CA).

### Statistical analysis

The trial's primary objective was to determine the efficacy of LI85008F in reducing body weight, BMI and normalizing lipid profile. A detailed statistical analysis was performed using SPSS software to evaluate the efficacy of LI85008F in comparison with the placebo group in terms of reduction in body weight, BMI, Lipid profile, and modulation of molecular biomarkers at baseline and days 14, 28 and 56 of treatment. Pair-wise changes were examined by carrying out a least significant difference test for all possible pairs. The significance of the effects of the treatment groups was compared by using one-way analysis of variance (ANOVA) followed by Tukey's multiple comparison tests. Results with P < 0.05 are considered statistically significant. For power calculations, the estimates for variability and assumed mean changes for each treatment group were based on results from a previous placebo-controlled clinical study [12]. We believe that an intervention that gives an average improvement of mean change ± 0.9 SD rather than mean change only will provide results of greater significance. Our trial is designed to have more than 90% power to detect a situation in which active treatment yields an improvement to at least mean change ± 0.9 standard deviation, under a conservative assumption, and we tested differences between groups in mean improvement using ANOVA (α = 0.05, two-sided). With 25 subjects per group, we would have at least 90% chance of observing one example of any side effect occurring in 10% or more of the subject population at a specific dosage.

## Results

### Baseline characteristics

The demographic variables, baseline disease characteristics and baseline outcome measures are provided in Table 2. Overall, the treatment group receiving LI85008F (900 mg/day, n = 21) and placebo (n = 20), were similar with respect to sex, age, body weight and Body Mass Index. The subjects were randomly distributed into placebo and treatment groups.

### Clinical efficacy

#### Reduction in body weight and BMI

**Figure 2:**
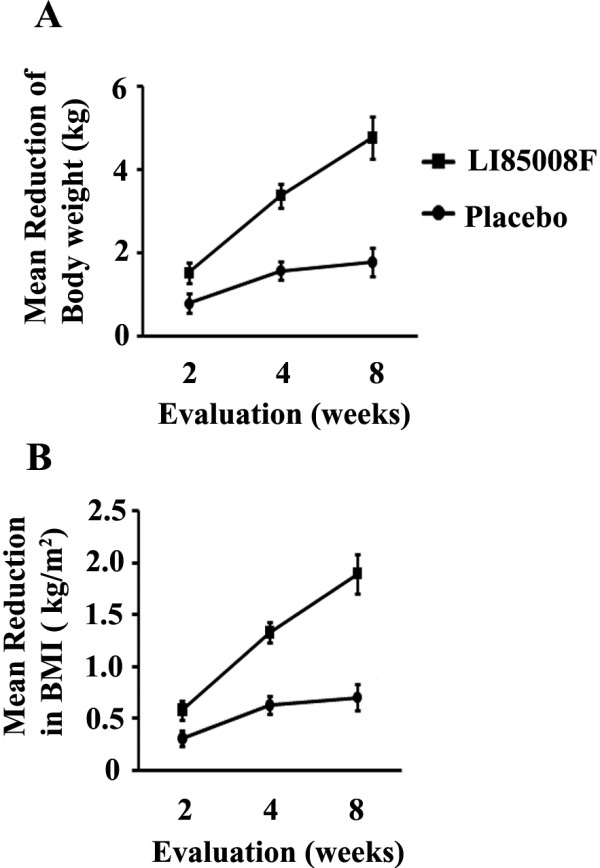
**LI85008F reduces changes in body weight and Body Mass Index in obese subjects.** Line diagrams represent mean reduction in body weight (**A**) and Body Mass Index (**B**) from baseline to 8 weeks in placebo (n = 20) and LI85008F (n = 21) supplemented groups, respectively. Values represent mean ± SE.

The present study evaluated the efficacy of LI85008F on weight loss, lipid profiles, serum adiponectin level in comparison with placebo group. During the study duration the trends in reductions of body weight and BMI, in comparison with the baseline evaluations are depicted in Figure 2 A and B, respectively. LI85008F supplementation for 8 weeks resulted in statistically significant body weight reduction (p < 0.001). Reduction in body weight in LI85008F group is 2.67 fold (166.56%) better than that in the placebo group. Significant reduction in body weight was observed at as early as 14 days in LI85008F supplemented subjects. LI85008F supplemented subjects lost 1.53 kg of their mean body weight in 2 weeks compared to 0.79 kg reduction in placebo group, which conferred a 92.76% better efficacy in body weight reduction (Table 3). LI85008F supplementation for 8 weeks resulted in statistically significant reduction of BMI (Table 3). Reduction in BMI in LI85008F group is 2.69 fold (169.1%) better than placebo group. Significant reduction in BMI was observed at as early as 14 days in LI85008F supplemented subjects. LI85008F supplementation provided 1.88 fold better reduction in BMI (0.585 kg/m²) compared to the placebo group (0.310 kg/m²) in 2 weeks.

**Table 3:** Changes in Physical Parameters from baseline to 8 weeks in the Placebo and in LI85008F supplemented group

**Measure**	**Study Period**	**Placebo (x ± SE)**	**P value**^**a**^	**LI85008F (x ± SE)**	**P value**^**a**^	**P value**^**b**^
Body Wt. (Kg)	Baseline	84.97 ± 2.80	< 0.001	86.16 ± 2.75	< 0.001	-
	8 week	83.18 ± 2.82		81.40 ± 2.69		
	Change	1.79 ± 0.34	-	4.76 ± 0.51	-	< 0.001
Body Mass Index (kg/m²)	Baseline	33.00 ± 0.73	< 0.001	34.41 ± 0.74	< 0.001	-
	8 week	32.30 ± 0.74		32.51 ± 0.75		
	Change	0.70 ± 0.13	-	1.90 ± 0.19	-	< 0.001
Waist Circumference (cm)	Baseline	108.59 ± 1.99	< 0.001	106.68 ± 1.67	< 0.001	-
	8 week	97.35 ± 2.53		94.16 ± 2.17		
	Change	11.24 ± 1.95	-	12.52 ± 1.83	-	0.6384
Hip Circum (cm)	Baseline	119.51 ± 1.50	< 0.001	117.99 ± 1.67	< 0.001	-
	8 week	110.68 ± 1.85		108.07 ± 1.65		
	Change	8.83 ± 0.99	-	9.92 ± 1.34	-	0.0934
Waist-Hip Ratio (WHR)	Baseline	0.91 ± 0.01	<0.05	0.91 ± 0.01	<0.05	-
	8 week	0.88 ± 0.01		0.87 ± 0.02		
	Change	0.03 ± 0.01	-	0.04 ± 0.01	-	>0.05
Systolic B.P. (mmHg)	Baseline	120.40 ± 3.40	0.4341	123.33 ± 2.87	0.4094	-
	8 week	118.00 ± 3.04		126.19 ± 2.80		
	Change	2.40 ± 2.93	-	−2.86 ± 3.39	-	0.2618
Diastolic B.P. (mmHg)	Baseline	76.20 ± 1.95	0.7388	80.00 ± 2.29	0.5614	-
	8 week	75.50 ± 1.53		81.43 ± 2.10		
	Change	0.70 ± 2.02	-	−1.43 ± 2.42	-	0.2146
Heart Rate (Beats/mim)	Baseline	73.20 ± 0.62	0.5953	74.29 ± 0.92	0.6676	-
	8 week	74.00 ± 1.34		74.95 ± 0.91		
	Change	−0.80 ± 1.45	-	−0.67 ± 1.53	-	0.4006

^a^ Difference within the group (baseline vs. final evaluation) using paired *t*-test.

^b^ Difference between changes (derived from baseline minus final evaluation) in placebo and LI85008F group using unpaired *t*-test.

#### Waist-hip ratio

In comparison with the placebo, better reduction of waist circumference and hip circumference was observed in LI85008F supplemented group. LI85008F supplementation reduced the waist and hip circumferences by 10.7% and 19.3% respectively compared to placebo group. Interestingly, the treatment group showed 33.96% more reduction in waist-hip ratio compared to placebo (Table 3).

#### Improvement in serum adiponectin

**Figure 3:**
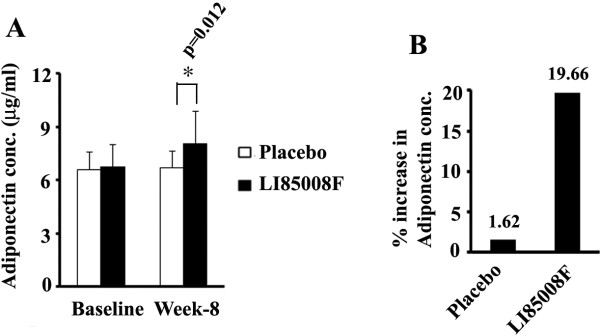
**LI85008F increases in serum Adiponectin.** Bar diagram represents the serum adiponectin concentration (mean ± SE) of subjects supplemented with LI85008F and placebo at the baseline and after 8 weeks. At the end of the study, LI85008F confers significant (p = 0.012) increase in adiponectin level in comparison with placebo (**A**). Bar diagram indicates comparative increase in serum adiponection concentration of placebo and LI85008F supplemented subjects after 8 weeks in comparison with the baseline (**B**).

LI85008F supplementation resulted in 21.26% (p = 0.012) increase in serum adiponectin concentration compared to the placebo group at the end of the study (Figure 3A). Further, it is interesting to note that 19.66% increase in serum adiponection concentration was observed in LI85008F group at week 8 in comparison with the baseline, in contrast, the placebo group showed only 1.62% increase in adiponection concentration (Figure 3B). This observation suggests the reduced fat store in LI85008F supplemented subjects. Interestingly, it is also suggestive that LI85008F might be a potential candidate for improving insulin sensitivity and cardiovascular health in obese humans.

#### Modulation of key biochemical parameters of fat metabolism

Reduction in serum glucose (17.0%), triglycerides (16.43%) and LDL/HDL ratio (12.6%) was observed in LI85008F treated group compared to baseline (Table 4). Placebo resulted in only 0.95%, -0.55% and 6.0% changes, in glucose, triglycerides and LDL/HDL ratio, respectively in comparison with the baseline observations. However, we did not measure the blood glycosylated hemoglobin (HbA1c) content of the study subjects. The results are summarized in Table 4.

**Table 4:** Changes in serum fat metabolic markers from baseline to 8 weeks in the Placebo and in LI85008F supplemented group

**Parameter**	**Study Period**	**Placebo (x ± SE)**	**P value**^**a**^	**LI85008F (x ± SE)**	**P value**^**a**^	**(P value)**^**b**^
Cholesterol (mM/L)	Baseline	5.33 ± 0.23	0.1176	5.58 ± 0.25	0.6479	-
	8 week	5.64 ± 0.22		5.48 ± 0.24		
	Change	−0.32 ± 0.19	-	0.09 ± 0.20	-	0.149
HDL-Cholesterol (mM/L)	Baseline	2.18 ± 0.05	0.5812	2.12 ± 0.08	0.2192	-
	8 week	2.14 ± 0.05		2.23 ± 0.07		
	Change	0.04 ± 0.07	-	−0.11 ± 0.09	-	0.11
LDL-Cholesterol (mM/L)	Baseline	9.03 ± 0.28	0.0925	9.21 ± 0.29	0.0005	-
	8 week	8.41 ± 0.28		7.67 ± 0.28		
	Change	0.62 ± 0.35	-	1.53 ± 0.37	-	0.0834
Triglycerides (mM/L)	Baseline	0.33 ± 0.01	0.8148	0.32 ± 0.02	0.0389	-
	8 week	0.33 ± 0.01		0.29 ± 0.01		
	Change	0.00 ± 0.01	-	0.03 ± 0.02	-	0.063
Glucose (mM/L)	Baseline	6.98 ± 0.61	0.8726	6.88 ± 0.35	0.0033	-
	8 week	6.91 ± 0.57		5.93 ± 0.27		
	Change	0.07 ± 0.41	-	0.94 ± 0.28	-	0.0669

^a^ Difference within the group (baseline vs. final evaluation) using paired *t*-test.

^b^ Difference between changes (derived from baseline minus final evaluation) in placebo and LI85008F group using unpaired *t*-test.

#### Adverse events

During the course of the 8 weeks study period, there were no major adverse events reported. However, some minor adverse events such as gastric irritation, abdominal pain and back pain were reported by few subjects. These minor events were distributed evenly between the placebo and treatment groups.

#### Dropouts

Four subjects from the active (LI85008F) treatment group were excluded because of their non-availability during the entire study duration and five subjects from the placebo were excluded due to non compliance to the study formulation. No subject was dropped out of the study as a result of an adverse event.

#### Modulations of adipokines in *3T3-L1* adipocytes

**Figure 4:**
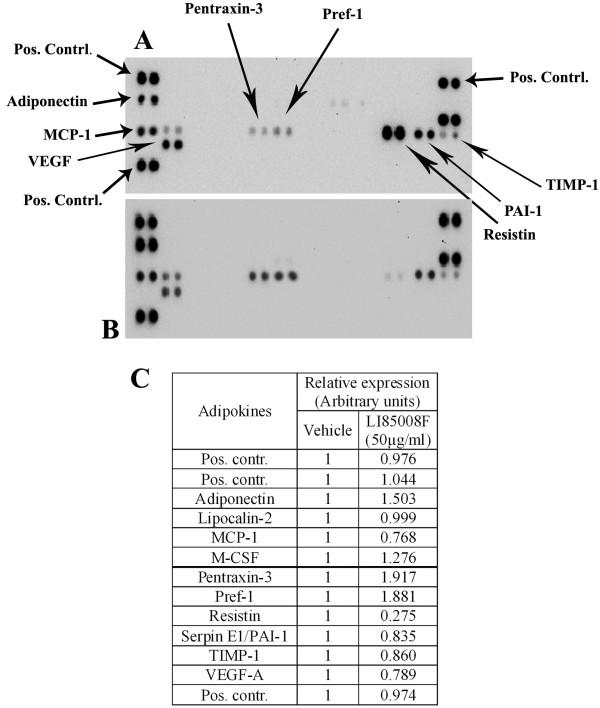
**LI85008F modulates adipokine in 3T3-L1 adipocytes.** Images of chemiluminescent reaction spots on adipokine profiler membranes representing various adipokines expressions in 3T3-L1 cells treated with vehicle (0.1% DMSO) and 50 μg/ml of LI85008F in **A** and **B**, respectively. Arrows indicate the adipokine spots on the membranes. The comparative expressions of adipokines (in arbitrary units) obtained from densitometric analysis of the chemiluminescent reaction spots are tabulated in Table (**C**).

Figure 4 A and B depict images of chemiluminescent reaction spots which represent various adipokines expressions in 3T3-L1 cells treated with vehicle (0.1% DMSO) and 50 μg/ml of LI85008F, respectively. The comparative analysis on the adipokines expressions reveals that LI85008F up regulates Adiponectin, Pentraxin-3, Pref-1; and down regulates MCP-1, Resistin, PAI-1 in 3T3-L1 adipocytes (Figure 4C). These adipokines are involved in the regulation of adipogenesis process and insulin sensitivity linked with obesity [13-19]. These observations suggest that the herbal formulation LI85008F modulates several key factors of adipogenesis that include adipocytes differentiation and insulin resistance linked with obesity.

## Discussion

Currently, there is an increasing global trend to depend on the plant based traditional therapies for the management of overweight and obesity due to various reasons such as adverse effects, habit-forming effects and high cost of the pharmaceutical drugs. Ayurveda, the traditional Indian Medicinal System, has long been known as the richest database of medicinal plants indicated for their uses in various disorders such as inflammation, pain, diabetes, obesity, hepato-toxicity, allergy, amnesia, etc. With an intention to develop a novel, safe and cost-effective herbal composition for the management of obesity in humans, we screened a panel of extracts prepared from over two hundred Indian medicinal plants for their anti-obese properties in mouse 3T3-L1 adipocyte models. We assessed the inhibition of adipogenesis and ability to enhance lipid breakdown i.e. lipolysis. From the screening assays, we selected several plant extracts including *Moringa olifera, Murraya koeingii*, *Curcuma longa* for their anti-adipogenesis activities. Furthermore, to achieve novel compositions with synergistic anti-adipogenesis efficacies, we combined several extracts in different ratios. It was found surprisingly that the composition (LI85008F) containing extracts of *M. olifera, M. koeingii*, *C. longa* at 6:3:1 ratio exhibited synergistic inhibition of adipogenesis in 3T3-L1 adipocytes*.* Further, a battery of *in vitro* experiments indicated that the basic approach to the anti-obesity properties of LI85008F is bi-directional; (i) it inhibits the formation of fat cells and intra-cellular fat accumulation and by impairing differentiation of pre-adipocyets to mature adipocytes, (ii) it decreases the fatty mass of adipocytes by increasing break-down of the intracellular lipid depot. Interestingly, the formulation LI85008F showed significant anti-obese efficacy in high fat diet induced obesity in Sprague Dawley rats (unpublished observation). Previously, we demonstrated a broad spectrum safety of LI85008F in appropriate animal models [10]. In this current double blind, randomized, placebo controlled clinical study (clinical trial registration no. IRCTN37381706) we have further assessed the anti-obesity efficacy of this novel herbal formulation. This manuscript presents the outcome of the present clinical study obtained by the intervention with the formulation, LI85008F.

Body weight gain and increase in BMI are the key clinical features of obesity. Previously, in other clinical studies, a number of herbal formulations such as *Glycine max* extract, *Camellia sinensis* extract, *Cissus quadrangularis* formulation (Cylaris) have been shown to reduce body weight and BMI in obese humans [20-22]. As shown in Figures 2 and 3, reductions of body weight and BMI are significant in subjects supplemented with LI85008F. The reduction of body weight and BMI by 166.6% and 169.1%, respectively following supplementation of LI85008F formulation for 8 weeks, strongly indicate its efficacy for weight management in obese humans. These anthropometric parameters are particularly important because these are the key determinants for obesity. Waist-hip ratio (WHR) is generally considered as a surrogate measure for abdominal visceral fat [23,24]. In the present study, interestingly LI85008F demonstrated 33.96% better reduction in WHR compared to placebo, further supporting its anti-obesity potential in obese humans.

Adiponectin is exclusively secreted from adipose tissue into the blood stream [15]. This protein hormone modulates a number of metabolic processes, including glucose regulation and fatty acid catabolism. Serum level of this hormone is inversely correlated with body fat percentage in human adults [25]. Significant number of studies indicates that reduced level of circulatory adiponectin may play a role in pathogenesis of obesity and type 2 diabetes [13,14,26]. We hypothesized that the supplementation of LI85008F could improve the circulating adiponectin level in obese humans. In our study, interestingly we observed that 8 weeks of LI85008F supplementation provided 21.26% (p = 0.0065) increase in serum adiponectin level in comparison with the placebo group (Figure 3). This observation supports our hypothesis and provides the molecular basis of its efficacy as a potent body fat reducing herbal formulation in obese humans. Interestingly, this weight loss formula might also be effective in the treatment of other symptoms associated with metabolic syndrome like insulin resistant type 2 diabetes in humans.

The modulation of certain key serological parameters related to obesity and fat metabolism (Table 4) by LI85008F formulation is correlated with reduction in body weight. We observed that the reduction in serum glucose, LDL/HDL ratio, and triglycerides followed a pattern similar to weight loss over the 8-week trial period. The reduced level of circulating lipids i.e. triglycerides and LDL/HDL ratio reflects improved status of fat metabolism and reduced stored fat in the body. In addition, the reduction of serum LDL/HDL ratio also implies a possible reduction in the risk of atherosclerosis and coronary heart disease [27].

Earlier we demonstrated in 3T3-L1 adipocytes that the possible mode of action by which the herbal formula LI85008F exerts its anti-obesity effect [9]. Further, in the present study the adipokine profiling in 3T3-L1 adipocytes reveals that LI85008F modulates several key factors involved in adipogenesis or lipogenesis process via adipocytes differentiation. The most interesting observation in this study is that the up regulation of adiponectin and pref-1 protein, and concurrently, down-regulation of resistin and PAI-1 in LI85008F treated cells. Increase in adiponectin level in LI85008F treated cells indicates reduced cellular adiposity and improvement in insulin sensitivity [28]. Interestingly, this observation from *in vitro* experiment is in agreement with the finding obtained from the clinical study, where LI85008F supplemented subjects demonstrated higher serum adiponectin level in comparison with the placebo. Preadipocyte factor-1 (Pref-1) is highly expressed in 3T3-L1 preadipocytes. Previous studies demonstrated that pref-1 null mice exhibited enhanced adipogenesis and also suggested the proposed role of pref-1 as a negative regulator of the adipogenic process [16,17]. Adipokine profiling study demonstrated enhanced expression of pref-1 in LI85008F treated cells, which suggests an inhibition of adipogenic differentiation process in 3T3-L1 adipocytes. Resistin is a secretory protein, produced by adipocytes, it impairs glucose tolerance in vivo and reduces glucose uptake by antagonizing insulin action in adipocytes *in vitro*[18]. Our observation indicates that LI85008F treatment drastically reduces resistin expression in 3T3-L1 adipocytes. This suggests the herbal formula has potential benefit in improving the insulin sensitivity. Taken together, our observations on adipokine profiling study in 3T3-L1 cells suggest that the herbal blend LI85008F not only modulates the key adipogenic factors to reduce adiposity but also modulates important adipocyte related proteins which regulate insulin sensitivity.

Herbal products are natural and are generally considered as safe. Above all, the ingredients of this anti-obese formulation i.e. *M. olifera, M. koeingii* and *C. longa* have long been known to be safe for human consumption. There are hundreds of years of history for usage of these individual plants as food ingredients in Indian sub-continental cuisine. As such, no major ill effect or adverse side effects are anticipated from the use of LI85008F. Although, some participants in the study reported some mild adverse effects, they were distributed evenly between the placebo and the treatment groups. The clinical evaluation of the biochemical safety parameters in blood and urine did not show any adverse results for the consumption of LI85008F. Therefore, in tune with our hypothesis, the current clinical study further provides support that the weight loss formula LI85008F is safe for human consumption. However, we consider the limitations of the current clinical study. A study design for long term anti-obese efficacy of LI85008F formulation with larger group sizes is warranted. In addition, it would also be interesting to assess the bioavailability of this herbal formulation in a suitable animal model. Some of the major phytochemical markers present in LI85008F (Adipromin) are Quercetin-3-O-glucoside, mahanine and curcumin. Bioavailability studies addressing quantitative estimation of these marker compounds in circulation of Adipromin fed rats are in pipeline.

## Conclusion

This natural weight loss formula LI85008F, composed of *Moringa olifera, Murraya koeingii, Curcuma longa* extracts, administered at a dose of 900 mg daily to the obese humans results in significant reduction in body weight and improvement of a critical biomarker associated with type 2 diabetes and obesity. It has also shown efficacy in the control of serum triglyceride concentration and LDL/HDL ratio and fasting blood glucose levels. In addition, analysis of various safety parameters related to blood and urine; and perusal of adverse events observed during the study indicate that this herbal formulation is safe for human consumption.

## Competing interests

This study is funded by Laila Impex R&D Center, India. KS, TG and KVA are employees of Laila Impex R&D Centre, Vijayawada, India. AM is an employee of ASRAM, Eluru, India. KM is an Ayurvedic Physician at Suraksha health village, Vijayawada, India. KVSS is a Professor in Department of Statistics, SV University, Tirupati, India. Authors declare that there is no conflict of interest in conducting the study and publishing the data.

## Authors’ contributions

KS contributed to the design of the project and data analysis, and was primarily responsible for writing the manuscript. KVA contributed to the design of the project and data analysis, subject recruitment and management, data collection and writing of manuscript. AM worked with subjects to obtain informed consent, conducted clinical evaluations, took samples and evaluated therapeutic response of LI85008F. TG contributed in the development of the formulation and coordinated the study. KM associated with study as a consultant Ayurvedic Physician. KVSS is a consultant statistician and contributed in clinical data analysis. All authors read and approved the final manuscript.
